# Strategies for maximizing consent rates for child dental health surveys: a randomised controlled trial

**DOI:** 10.1186/1471-2288-13-108

**Published:** 2013-09-04

**Authors:** Anne-Marie Glenny, Helen V Worthington, Keith M Milsom, Eric Rooney, Martin Tickle

**Affiliations:** 1 School of Dentistry, The University of Manchester, Manchester M13 9PL, UK; 2 Cumbria & Lancashire Centre Public Health England, Preston, UK; 3 Cheshire & Merseyside Centre, Public Health England, Chester, UK

**Keywords:** Consent, Epidemiological survey, Dentistry, Randomised controlled trial

## Abstract

**Background:**

Poor response rates can jeopardise the validity of the findings of epidemiological surveys. The aim of this study was to undertake a randomised controlled trial to determine the effectiveness of different strategies for maximizing parental consent rates for dental health surveys of young children.

**Methods:**

The trial took place within the 2007/2008 NHS Epidemiological Dental Health Survey of 5-year-old children in the North West of England. Schools were randomised to one of five interventions: multiple letters to parents; promoting the research by providing additional information to parents and children; a financial incentive to the school; a financial incentive to the school administrator plus direct mailing to parents; and a control intervention comprising of usual practice, that is a single letter home to parents via the children.

**Results:**

A total of 335 schools (11,088 children) were recruited. The mean percentage consent rates ranged from 47% (financial incentive to school administrator plus direct mailing) to 63% (multiple letters). Pair-wise comparisons indicated that the multiple letter group had a statistically significantly greater consent rate than the financial incentive to the school administrator plus direct mailing group and promoting the research by providing additional information group, but was not statistically significantly different from the financial incentive to the school group and the control group.

**Conclusions:**

There was little evidence to show that any of the five interventions made a significant difference to consent rates when compared to the control group. Financial incentives to schools were less effective than multiple reminder letters to parents. Trials should be built into surveys to test different interventions, in different contexts to expand the evidence base for improving consent rates in health surveillance programmes.

## Background

Since 1985 a series of annual National Health Service (NHS) surveys of children’s dental health have been co-ordinated by the British Association for the Study of Community Dentistry (BASCD) [1,2] and more recently by the North West Public Health Observatory. In England NHS Primary Care Trusts (PCTs) have a statutory duty [3] to carry out regular oral health surveys to facilitate:

• the assessment and monitoring of oral health needs,

• the planning and evaluation of oral health promotion programmes,

• the planning and evaluation of the provision of primary and specialist dental services, and

• the monitoring and reporting of the effect of water fluoridation programmes

This epidemiological programme uses nationally standardised sampling strategy, methodology and diagnostic criteria [4,5] for clinical examinations undertaken in a school setting.

Following publication of guidance from the Department of Health in May 2006, [6] there was a fundamental change in the process of obtaining parental consent for the participation of children in these surveys. Historically, a process of ‘passive’ consent had been used, whereby parents or guardians were provided with a written explanation of the proposed survey and were given the opportunity to withdraw their child from the survey. Following the change in national guidance passive consent was no longer acceptable and those conducting school dental surveys are now required to obtain ‘active’ consent, that is parents of all children who are not competent are required to provide written, informed consent prior to examination. Moving to active consent gave rise to concerns about the possibility of reduced numbers of children participating in the surveys and an increased risk of non-response bias, potentially jeopardising the validity and representativeness of the epidemiological data collected [7-9]. A concern that seems to have some foundation, as the response rate by PCT in the 2007/2008 NHS Dental Health Survey of 5 year-olds ranged from 24.3 to 90.3 per cent. The report of this survey stated ‘*In previous surveys the response rates of 75.0% and above have been readily achieved and considered by BASCD to provide sufficient confidence to enable publication and comparison with the results of previous surveys. In England during 2007/08, only 66.8% of the drawn sample were included in the final analysis therefore national level comparisons with previous surveys cannot be made with confidence.*’ [10]

It is therefore important to evaluate interventions to increase response rates for these surveys. However the issue of poor consent rates increasing the likelihood of introducing bias into survey results is not just an issue for dental or NHS surveys, this is a much broader issue which affects any surveillance or research programme involving children in the school setting. Monetary and non-monetary incentives to increase response rates to mail surveys have been described in the literature, and monetary incentives have shown to be effective in increasing response rates [11,12]. Monetary incentives that have been paid upfront (sent initially with the questionnaire) have been demonstrated to be more effective than payments made contingent on receiving a response [13]. However, responding to mail surveys is very different to providing consent for a survey that includes a clinical intervention such as an epidemiological examination. A systematic review exploring strategies to improve recruitment to research studies highlights that current research evidence is unable to predict the effect most interventions will have on recruitment [14]. However, the review concluded that it would be beneficial to try to reproduce the results of interventions that were found to be effective in other contexts, such as interventions involving financial incentives [15], and provision of additional information about surveys to prospective participants. These studies relate primarily to strategies to improve participation in postal surveys. However, research in a school setting, and obtaining consent from parents to involve their children in research have specific features that researchers need to be cognisant of when seeking to consent children into school-based studies. A recent review of strategies [16] to obtain active parental consent in school-based research identified 1) promotion of the research to school principals, teachers, parents and students; 2) dissemination of study information using methods allowing direct contact with parents (i.e. telephone or face-to-face); 3) provision of incentives to teachers, students and at a class level; 4) making reminder contacts; and 5) having a member of the research team co-ordinate and closely monitor the recruitment process as strategies which may be effective in enhancing participation rates. These strategies may or may not be possible in different contexts, the authors of the review recommended that further randomised controlled trials of strategies to improve consent in school-based research studies are required to strengthen the evidence base.

A randomised controlled trial was undertaken to assess the effectiveness of different strategies, including financial incentives and the provision of additional information, on the recruitment of young children into dental health surveys.

## Methods

The trial took place opportunistically within the 2007/2008 NHS Dental Health Survey of 5 year-old children in the North West of England. These surveys have been conducted since the mid 1980s and are a statutory requirement of PCTs [3] and so schools have a long history of working with NHS personnel to undertake these surveys. School randomisation was used rather than individual randomisation of children to avoid contamination between groups, as consent is usually organised centrally by the school and some of the interventions to be tested were applied to schools rather than to individuals. The unit of randomisation and analysis was the school and we have therefore not described this study as a cluster randomised trial. There are over 2400 schools across 24 PCTs within the North West of England. Seven PCTs were invited to participate: Halton & St Helens PCT, Warrington PCT, Western Cheshire PCT, Knowsley PCT, Liverpool PCT, Blackburn & Darwen Teaching PCT, East Lancashire PCT.

Eligibility was determined by the national NHS dental epidemiological survey protocol [4,5]. The eligibility criteria in the protocol for children were that they should be attending state maintained primary schools and have reached the age of five, but have not had their sixth birthday on the date of examination. Only state maintained schools were included in the study and the schools identified children in the relevant age cohort according to their year group. The inclusion criteria for the trial were defined as any school, with 20 or more 5-year-old children participating in the Dental Health Survey within these PCTs.

### Interventions

Schools were randomized to one of five arms:

*Financial incentive to school administrator and direct mailing (FISA*&*DM)* – each school was provided with stamped, named envelopes, containing a standard information leaflet and standard consent form, to be addressed by the school and posted to the parents of the selected children. Parents were asked to return the consent form to the school. In each school the person responsible for the management of the consent letters was to receive a £50 store voucher if the school achieved a consent rate of 75% or above. If the school didn’t think it was appropriate that an individual received the vouchers then the school was given the voucher.

*Financial incentive to the school (FIS) –* named envelopes, containing the standard consent letter for each child selected to be surveyed, were distributed by the school and sent to the child’s parent/guardian via the child. Each school was given £4.00 for each selected child whose parents consent to the survey.

*Promotion of the survey* – the head teacher of each school was provided with information about the importance of oral health and the dental survey, to be presented at a school assembly (a daily meeting through which information is conveyed to pupils by the headteacher). In addition each parent was given a ‘glossy leaflet’ that described the importance of oral health along with the standard consent letter. These were distributed via the children who returned the consent forms to the school.

*Multiple letters –* named envelopes, containing the standard consent letter for each child selected to be included in the survey, were distributed by the school and sent to the child’s parent/guardian via the child. Two weeks after the consent forms were sent out, the schools were contacted to find out how many had been returned. A second letter was prepared for non-responders and sent to the school for distribution. Not less than 14 days later each school was contacted again to establish how many forms had been returned and to identify the non-responders. We took advice from the schools with regard to sending a second reminder letter to remaining non-responders and the consensus was that this would add little value. Therefore all schools in this arm ceased any further attempts to increase consent rates after a single reminder letter was sent to parents.

*Control arm* – (usual practice) named envelopes, containing the standard consent letter for each child selected to be included in the survey, were distributed by the school and sent to the child’s parent/guardian via the child. No further contact was made.

The interventions chosen were dictated by what was pragmatically possible in incentivising responses to school surveys funded by the NHS in the UK. The literature suggests that direct payments are likely to be effective [13,15], however direct payments to parents and\or children is ethically problematical, as UK ethics committees would have concerns that direct payments, particularly to vulnerable groups such as young children, would potentially coerce children into participating in the study. Secondly given the scale of the NHS surveys and the numbers of children involved, direct payments to parents and/or children, would not be affordable. PCT staff with responsibility for administrating the NHS survey were briefed in detail about the trial. A training day was undertaken, led by members of the research team to provide an opportunity for PCT staff to discuss the different interventions used in the trial.

### Outcomes

The primary outcome measure was consent rate, defined as the proportion of children whose parents provided consent for them to participate in the survey. It should be noted that consented children might not participate in the survey, for example if they are ill on the day the survey examination is scheduled for their school. The consent rate was measured by a count of the returned consent forms. The denominator was an up-to-date list of children currently on the school register; the schools provided this.

### Sample size

The sample size was designed to detect a difference in consent rates between 55% and 70%, assuming a common standard deviation of 20%. As all pairwise comparisons were to be made (5 groups, 10 comparisons), for an overall alpha value of 0.05 the p-value for each comparison was 0.005. A trial with 60 schools in each arm would have 90% power to detect this difference. For logistical purposes, the trial aimed to recruit all schools in the identified PCTs meeting the inclusion criteria.

### Randomisation

Eligible schools were stratified by (i) PCT, (ii) size of school (medium <50 children aged 5 years; large ≥50 children aged 5 years) and (iii) socioeconomic status (schools were placed in quintiles according to Index of Multiple Deprivation (IMD) score [17] of the Super Output area in which each school was located for randomization to the five intervention arms according to a computer generated sequence. The randomization and allocation to intervention groups were undertaken centrally by a third party (Clinical Trials Unit, Christie Hospital, Manchester). The trial investigators were unaware as to which group a school had been randomized to until the school was allocated to the group. Due to the nature of the interventions it was not be possible to blind schools to the intervention they received.

### Statistical analysis

The outcome measure was the percentage of children in each school who consented to participate in the survey. This outcome was thought to be normally distributed across schools and we expected parametric methods on continuous data to be appropriate. Initially a multiple linear regression model for the dependent variable ‘percentage consenting’ was fitted with the number of consent forms sent out by the school, and study group as dependent variables to investigate if the number of consent forms was a significant covariate. Following this analysis a simple one-way ANOVA was conducted on the percentage consenting per school using the post hoc Bonferroni criterion to make pair-wise comparisons between groups, and this was used to present the results. A further analysis was conducted including PCT and quintile of socio-economic status of the school (based on the Super Output area in which the school was located) to see if the inclusion of these factors changed the primary analysis.

Before commencing the trial, ethical approval was obtained from the University of Manchester. Following advice from the local research ethics committee, NHS ethical approval was not required. In line with research governance requirements, approval was obtained from the Research and Development offices of participating PCTs.

## Results

Six PCTs (located over 8 local government authorities) participated in the trial. A total of 335 schools (11,088 children) were recruited. The number of schools in each PCT ranged from 39 to 81, with 80 schools classified as large and 255 classified as medium sized schools (Table 1). Over 40% of the schools were placed in the 5^th^ quintile (least affluent) with regard to IMD scoring (based on National data).

**Table 1:** Number of schools randomized to each intervention by PCT, size and IMD quintile

**PCT**	**FISA**&**DM**	**FIS**	**Promotion of the survey**	**Multiple letters**	**Control**	**Total N (%)**
Blackburn and Darwen	7	7	10	9	9	42 (12.5)
Halton and St Helens	11	17	18	14	12	72 (21.5)
Knowsley	13	11	6	9	13	52 (15.5)
Liverpool	19	14	14	16	18	81 (24.2)
Warrington	12	5	11	11	10	49 (14.6)
Western Cheshire	5	13	9	7	5	39 (11.6)
School size						
Medium	50	50	54	49	52	255 (76.1)
Large	17	17	14	17	15	80 (23.9)
IMD quintile						
1 (most affluent)	6	6	4	8	6	30 (9.0)
2	7	10	9	7	6	39 (11.6)
3	12	7	10	10	8	47 (14.0)
4	11	17	16	12	14	70 (20.9)
5 (least affluent)	31	27	29	29	33	149 (44.5)
Total N (%)	67 (20.0)	67 (20.0)	68 (20.3)	66 (19.7)	67 (20.0)	335

**Figure 1:**
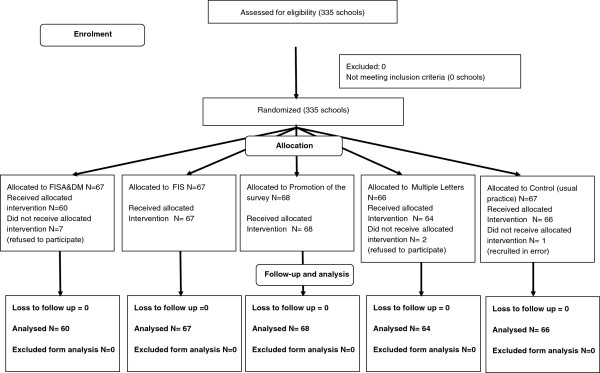
CONSORT diagram.

Of the 335 recruited schools, one school was recruited to the control group in error, and nine schools refused to participate in the trial (but continued to participate in the survey) once they knew the outcome of their allocation, leaving 325 schools for analysis. A CONSORT flow diagram (Figure 1) presents the number of schools randomized to each intervention and included in the analysis.

A multiple regression model for the dependent variable ‘percentage consenting’ indicated that the number of consent forms sent out by the school was not a significant covariate (p = 0.43), therefore a simple one-way ANOVA was conducted on percentage consenting using the post hoc Bonferroni criterion to make pair-wise comparisons between groups. The ANOVA F-ratio test indicated that there were differences in consent rates between the groups (F_4,320_ = 5.71, p < 0.001)

The mean percentage consent rates ranged from 47% in FISA&DM to 63% for Multiple letters (Table 2). Pair-wise comparisons (Table 3) indicated that the Multiple Letter group and the Promotion of the Survey group achieved statistically significantly greater consent rates than the FISA&DM group. However, neither the Multiple Letter group nor the Promotion of the Survey group were not statistically significantly different from any other intervention.

**Table 2:** Consent rates by intervention

	**FISA**&**DM**	**FIS**	**Promotion of the survey**	**Multiple letters**	**Control**
N	60	67	68	64	66
Mean consent rate (%) (95%CI)	47 (41 to 53)	54 (49 to 59)	58 (54 to 62)	63 (58 to 69)	57 (52 to 62)
Standard deviation	(22)	(20)	(17)	(21)	(20)

**Table 3:** Pairwise comparisons of mean percentage of consents per school by intervention group (Bonferroni criterion)

**Interventions**		**Mean difference**	**Standard error**	**p-value**	**95% confidence interval**
FISA&DM	FIS	−6.80	3.60	0.59	−16.93 to 3.34
	Promotion of the survey	−11.11*	3.57	0.02	−21.21 to −1.01
	Multiple letters	−16.65*	3.63	<0.001	−26.89 to −6.40
	Control	−10.02	3.60	0.06	−20.19 to 0.15
FIS	Promotion of the survey	−4.32	3.47	1.00	−14.13 to 5.50
	Multiple letters	−9.65	3.53	0.06	−19.82 to 0.12
	Control	−3.22	3.50	1.00	−13.11 to 6.67
Promotion of the survey	Multiple letters	−5.53	3.51	1.00	−15.46 to 4.40
	Control	1.10	3.50	1.00	−8.76 to 10.95
Multiple letters	Control	6.63	3.54	0.62	−3.38 to 16.63

*statistically significant at the 0.05 level.

A further analysis of variance including PCT and the quintiles of socio-economic status of the school as factors was conducted and the results of the pairwise comparison were compared to those presented in Table 2. They were similar; the only difference found was that the control group had a significantly greater consent rate than the FISA&DM group (P = 0.003).

## Discussion

There is a concern that the move to active consent has had a detrimental impact on the numbers of children participating in the NHS Dental Epidemiology Programme [10]. This concern has been reinforced by this study, as all of the intervention groups and the control group had response rates which could increase the risk of bias and thus affect the validity of the results obtained. In this trial none of the interventions designed to improve consent out-performed usual practice (control), which was a simple letter sent home via the child. However, multiple letters and promotion of the survey within the school, were significantly better than a financial incentive to the school administrator responsible for organising consent. A weakness of the trial was that individual incentives to children or parent were not tested for ethical and financial reasons. In this NHS context this approach would be unaffordable because of the numbers of participants involved in this national surveillance programme.

It has been suggested that studies that are unable to recruit sufficient participants might miss clinically important information and, therefore, be an inappropriate use of resources [18]. For example, in a study investigating the impact of consent on prevalence of dental caries in 12-year-old children examined in school, 414 potentially eligible children were identified. Active consent was used for recruitment purposes. Approximately one third of consent forms were not returned, and an additional 14% of those consented refused to participate in the study. Less than 50% of children were examined, which could weaken the findings of the study [7]. Low response rates in surveys can bias the findings due to systematic differences in those that agree to participate and those that do not [8,9]. It has been suggested in the past that such biases are reduced in surveys with response rates of 70% or above [19]. However, there is a growing recognition that studies with low response rates are not automatically subject to bias. [8,9,20,21] Bias will only be present if there are systematic differences between consenting and non-consenting individuals. Methods exist to detect and to estimate the effects of non-response bias [8,22,23] but these methods can’t accurately quantify non-response bias. The best way to reduce the risk of non-response bias is to increase response rates; this was the main reason for undertaking the trial. Unfortunately none of the interventions tested were significantly better than the control; a one shot letter to parents delivered by children.

There are potentially many strategies for improving consent rates to healthcare studies. This trial focused on interventions that had previously been found to be effective [14,16]. However, despite previous studies [16] showing financial incentives and promotion of the research to be effective at increasing recruitment rates, the results of this study do not support this finding. It must be pointed out that this study was a pragmatic trial and the interventions tested were what would be affordable or pragmatically possible in NHS disease surveillance programmes. We made no attempt to independently verify adherence of schools to the protocol in each arm of the trial. This would have been very expensive and might not have been acceptable to schools, which in itself could have adversely affected recruitment rates. In the operational delivery of all of these interventions we could not insist on, or independently verify strict adherence to protocols because these NHS surveys are reliant on the goodwill of schools. The fieldwork team had excellent, long-standing relationships with the schools and we believe that the schools participating in the study were diligent and adhered to the intervention protocols, although we have no evidence to verify this belief. Adherence might be assured through formal contractual arrangements but this would increase costs and might discourage schools from participating. The pragmatic nature of the trial should be seen as a strength, as the findings reflect the outcomes that researchers can expect in operational surveillance programmes. However, a parallel qualitative study might have explained some of the findings and identified ways that the interventions could have been improved to increase consent rates. This approach could be a potential topic for further investigation. Multiple letters targeting non-responders, which is a long-standing and commonly used strategy in questionnaire surveys [24], were shown to produce a statistically significant higher consent rate (63%) than providing one form of financial incentive (47%). However, the consent rate achieved using multiple letters was not statistically higher than that in the control group (57%), suggesting that there is insufficient evidence to support a change in current recruitment strategies. Further evaluation of strategies, testing different interventions to increase consent rates is required. Such trials, like the one reported here, are easily incorporated into large, routine health surveillance surveys and the public health community should consider this approach when designing surveys to improve the evidence base to increase survey response rates. In addition, assessment of the extent of non-response bias should be undertaken to determine the generalizability of findings [23].

It is feasible that recruitment strategies are setting-specific, age-specific (this trial investigated obtaining parental consent, as the children in the study were not deemed to be competent to provide consent) and topic-specific; for example, it is feasible that the more invasive the procedure the more likely the consent rate will be low. So the results of this trial may not be generalizable to other settings, other age groups or studies in other fields of research. The data reported here show that 40% of schools in the PCTs included in the trial were in the most deprived quintile. This may cause some concerns about generalizability of the findings, however we see this as a positive aspect of this trial, as non-response is usually higher in disadvantaged populations than more affluent populations [21,22] and therefore it is important to evaluate interventions to increase consent to surveys in these high risk, low compliant populations. This sensitivity to context underlines the need to undertake many more trials in different populations and in different circumstances.

School-based surveys require strong co-operation from the schools involved in order to obtain lists of pupils, distribute consent forms and collect responses [7]. The NHS dental surveys have been conducted for over 20 years, during which time staff undertaking the surveys and local schools have established good working relationships. In such settings with this history, alterations to recruitment strategies may be less likely to influence consent rates. Even with good, well-established working relationships, as is the case for the PCTs and schools in this study, this did not result in response rates which would automatically give confidence in the validity of the survey results. There was large variation in response rate by school within each arm of the study, strongly suggesting that individuals responsible for obtaining consent within schools, or school policy had a significant effect on consent rates. The effect of how conscientious a school administrator is or the culture of a school has on the consent rates is very difficult to measure and beyond the scope of this trial but is something to be aware of in future studies.

### Implications for practice

This trial has broad implications for researchers and public health practitioners undertaking school-based research projects or surveillance programmes. Based on our findings of this trial those undertaking school-based surveys can maximise consent rates by providing schools with named envelopes, containing the standard consent form and standard information leaflet for each child selected to be surveyed. The letters should be distributed by the school and sent to the child’s parent/guardian via the child. Commissioners and providers of the UK dental epidemiology programme should continue to stress the importance of the survey for the planning and provision of future dental services and maintain good working relationships with the individual schools. Further evaluation of techniques to maximise consent rates needs to be undertaken, and designed into future surveys, possibly with parallel qualitative components. Increasing consent rates reduces the risk of non-response bias, but in addition to testing new interventions to increase consent, public health practitioners and epidemiologists should routinely employ methods to detect and estimate the effect of non-response bias in surveillance programmes.

## Conclusions

In conclusion there was little evidence to show that any of the five interventions, which are feasible to adopt routinely in school-based health surveillance surveys, made a significant difference on consent rates when compared to the control group. Financial incentives to schools were less effective than multiple reminder letters to parents. Ongoing research is needed to test methods of improving consent rates in school-based research and health surveillance programmes, and nested trials similar to the one reported here, but testing different interventions, should be built into future dental surveys, and surveys conducted to investigate other diseases and conditions.

## Competing interests

The authors declare that they have no competing interests.

## Authors’ contribution

AMG: Study conception and design, study management, interpretation of findings manuscript preparation, manuscript revision. HVW: Study statistician, study design, analysis design and completion, interpretation of findings, manuscript preparation, manuscript revision. KM: Study design, fieldwork management, manuscript preparation. ER: Study design, fieldwork support, manuscript preparation. MT: Study conception and design, study management, interpretation of findings manuscript preparation, lead on manuscript revision, corresponding author. All authors read and approved the final manuscript.

## Pre-publication history

The pre-publication history for this paper can be accessed here:

http://www.biomedcentral.com/1471-2288/13/108/prepub
